# miR-1236-3p inhibits invasion and metastasis in gastric cancer by targeting MTA2

**DOI:** 10.1186/s12935-018-0560-9

**Published:** 2018-05-01

**Authors:** Jia-Xiang An, Ming-Hui Ma, Chun-Dong Zhang, Shuai Shao, Nuo-Ming Zhou, Dong-Qiu Dai

**Affiliations:** Department of Gastroenterological Surgery, The Fourth Affiliated Hospital of China Medical University, Shenyang, 110032 China

**Keywords:** Gastric cancer, Metastasis, miR-1236-3p, MTA2, Epithelial–mesenchymal transition, Tumor suppressor gene

## Abstract

**Background:**

MicroRNAs deregulation are common in human tumor progression. miR-1236-3p has been reported to function as tumor suppressor microRNA in various malignancies. The aim of this study was to demonstrate the downregulated expression of miR-1236-3p in gastric cancer (GC) tissues and cell lines, and clarify its biological function in GC.

**Methods:**

Real-time polymerase chain reaction was used to measure the mRNA level of miR-1236-3p in GC. Dual luciferase assay was used to demonstrate that MTA2 was one of the candidate target genes of miR-1236-3p. Western blots were utilized to detect the protein levels. Cell function assays were also performed to determine the function of miR-1236-3p in GC.

**Results:**

miR-1236-3p expression, which was associated with lymph node metastasis, differentiation and clinical stage, was significantly reduced in GC tissues and cell lines. miR-1236-3p over-expression could inhibit GC cell proliferation, migration and invasion, and inhibition of miR-1236-3p expression had opposite effects. Furthermore, we demonstrated that MTA2 was a candidate target of miR-1236-3p, and miR-1236-3p over-expression significantly inhibited the process of epithelial–mesenchymal transition. We also found that miR-1236-3p could suppress the PI3K/Akt signaling pathway in GC cells.

**Conclusions:**

Our results suggest that miR-1236-3p functions as a tumor suppressor in GC and could be a promising therapeutic target for GC.

## Background

Gastric cancer (GC) is the second most frequent cause of cancer deaths in the world, and remains the type of cancer with the highest incidence in northeast Asia [1]. Despite a variety of strategies to improve the survival rate of patients with GC, the survival time of patients with advanced GC is still short [2–4], and effective therapies are limited [5, 6]. Therefore, identification of new early diagnostic biomarkers and development of new anticancer targeted therapies is imperative.

MicroRNAs (miRNAs) are a class of small, non-coding RNA species, approximately 19–25 nucleotides in length. They can bind to the 3′-untranslated region (UTR) of the target mRNA and promote or inhibit the expression of target genes at the post-transcriptional level [7, 8]. Moreover, they are also able to bind to the 5′UTR region and ORF (open reading frame) [9]. Studies have shown that many miRNAs function as tumor suppressors or oncogenes in cancer cells, and participate in various biological processes such as proliferation, differentiation, apoptosis, and metastasis [10, 11]. Dysregulation of numerous miRNAs, such as mir-122, mir-26a, and mir-200c, has already been identified in GC [12–14], which suggests that miRNAs could serve as a class of new molecular biomarkers for GC [15, 16].

Mir-1236-3p, an intronic miRNA, is located in the Chr6p21.33 and embedded within the intron of the NELFE gene. Accumulating evidence predicts that miR-1236-3p may act as a tumor suppressor gene, and downregulation of miR-1236-3p has been demonstrated in some cancers. In hepatocellular carcinoma, miR-1236-3p down-regulates alpha-fetoprotein (AFP), thus causing PTEN accumulation, which inhibits the PI3K/Akt pathway [17]. In high-grade serous ovarian carcinoma, miR-1236-3p represses cell migration and invasion abilities by targeting ZEB1 [18]. In renal cell carcinoma, miR-1236-3p directly targeted the p21 promoter, and increased miR-1236 expression inhibited cell proliferation, and decreased CDK4/6 and cyclin D1 expression [19]. Moreover, miR-1236-3p down-regulation was also reported in bladder cancer, lung cancer, and breast cancer [20, 21], however, its biological function in GC is remains unclear.

Through preliminary screening we found that miR-1236-3p is significantly down-regulated in GC, and may serve as a potential diagnostic biomarker and therapeutic target in GC. The purpose of this study is to investigate miR-1236-3p expression in GC and cell lines. The inhibition of miR-1236-3p expression promoted cell proliferation, migration, invasion, cell epithelial–mesenchymal transition (EMT) processes, and the PI3K/Akt signaling pathway. Furthermore, we showed that miR-1236-3p could directly target at MTA2 and regulate its expression. Thus, we demonstrated that miR-1236-3p functioned as a tumor suppressor in GC at least partly through inhibition of the EMT process and PI3K/Akt signaling pathway by targeting MTA2.

## Materials and methods

### Clinical samples

Eighty-three paired specimens of cancerous tissues and adjacent non-tumorous tissues were obtained from patients with GC who underwent surgery at the Cancer Research Institute of China Medical University (Shenyang, China) between 2013 and 2016. All specimens had been diagnosed with GC using histopathological confirmation. None of the patients underwent chemotherapy before surgery. Tissue samples were snap-frozen in liquid nitrogen after surgical removal. Informed written consents were obtained from all patients. The study was conducted in accordance with the Declaration of Helsinki, and the protocol was approved by the Ethics Committee of China Medical University. A summary of the clinicopathological data from the study is given in Table 1.

**Table 1:** Correlation between miR-1236-3p expression and clinicopathological features in 83 GC patients

Variable	Case number	miR-1236-3p expression		*P* value
		Low (n = 55)	High (n = 28)	
Age				0.644
< 60	24	15	9	
≥ 60	59	40	19	
Gender				0.306
Male	62	43	19	
Female	21	12	9	
Tumor invasion				0.520
T1–T2	23	14	9	
T3–T4	60	41	19	
Lymph node metastasis				*0.018*
Yes	53	40	13	
No	30	15	15	
Differentiation				*0.015*
Well/moderate	35	18	17	
Poor	48	37	11	
TNM stage				*0.003*
I–II	32	15	17	
III–IV	51	40	11	

The *P* values shown in italic are the statistically significant values (*P* < 0.05)

### Cell lines culture

The three human GC cell lines MKN-45, SGC-7901, MGC-803, and a normal human gastric cell line GES-1 were obtained from Typical China Academy Culture Collection Commission Cell Library (Shanghai, China). All cells were cultured in RPMI 1640 medium (Invitrogen, Carlsbad, CA, USA) supplemented with 10% fetal bovine serum (Gibco, BRL, UK) and were maintained at 37 °C in 5% CO_2_.

### RNA isolation and quantitative real-time PCR of miR-1236-3p

Total RNA was extracted using TaKaRa MiniBEST Universal RNA Extraction Kit (Takara, Dalian, China). Reverse transcription was performed with a Hairpin-it™ miRNA RT-PCR Quantitation Kit (GenePharma, Shanghai, China) at 25 °C for 30 min, 42 °C for 30 min, and 85 °C for 5 min. U6 RNA was used as an internal control. Real-time PCR was performed in an Applied Biosystems 7500 Real-Time PCR system (Applied Biosystems, Foster City, CA, USA) according to the manufacturer’s instructions with the following steps: 3 min at 95 °C; followed by 40 cycles of 12 s at 95 °C, and 40 s at 62 °C. The relative gene expression was calculated using the 2^−ΔΔCt^ method. The PCR primers for mir-1236-3p were 5′-CCAATCAGCCTCTTCCCCTT-3′ (Forward) and 5′-TATGGTTGTTCACGACTCCTTCAC-3′ (Reverse). The primers for U6 were 5′-ATTGGAACGATACAGAGAAGATT-3′ (Forward) and 5′-GGAACGCTTCACGAATTTG-3′ (Reverse).

### Quantitative real-time PCR of MTA2

MTA2 cDNA was reverse-transcribed according to the manufacturer’s instructions (Takara, Dalian, China). β-actin was used as the reference gene. Quantitative real-time PCR was carried out with SYBR Green (Solarbio, China) in a total volume of 20 μL using an Exicycler 96 Real-Time Quantitative Thermal Block (Bioneer, Daejeon, Korea). The reactions were incubated at 94 °C for 10 min; followed by 40 cycles of 94 °C for 10 s, 60 °C for 20 s, and 72 °C for 30 s. Melting curves were generated to confirm the specificity of the amplification. Experiments were repeated in triplicate. Relative gene expression was calculated using the 2^−ΔΔCt^ method. The primers for MTA2 were 5′-ATCATTACCAGCCACCCA-3′ (Forward) and 5′-CGATTATCAGATTCTCCCTC-3′ (Reverse). The primers for β-actin were 5′-CTTAGTTGCGTTACACCCTTTCTTG-3′ (Forward) and 5′-CTGTCACCTTCACCGTTCCAGTTT-3′ (Reverse).

### Cell transfection

The lentivirus-hsa-miR-1236-3p mimic, the lentivirus-hsa-miR-1236-3p inhibitor, and a miRNA negative control (NC) were purchased from Genechem (Shanghai, China). The miR-1236-3p mimic and the negative control were transfected into SGC-7901 cells, and the miR-1236-3p inhibitor and the negative control were transfected into MKN-45 cells.

For lentivirus infection, cells were incubated with lentivirus and 5 μg/mL polybrene for 24 h. After infection, cells were measured using a fluorescent inverted microscope. The efficiency of lentivirus infection was evaluated by real-time PCR.

### Cell proliferation assays

Transfected cells were grown on 96-well plastic dishes in normal culture medium. Then, Cell Counting Kit-8 (CCK-8) working solution (Keygen, Jiangsu, China) was added into the medium according to the manufacturer’s protocol. Cells were incubated with 10 μL/well of CCK-8 solution during the last 4 h of the culture. Then, a microplate reader was used to detect the absorbance of the wells at 0, 24, 48, and 72 h. Each experiment was performed three times.

### Wound-healing assays

For wound-healing assays, transfected cells were plated in six-well plates. When the cell confluence reached 90–100%, a linear scratch was created using a 200 µL pipette tip. The wounded monolayer was washed with phosphate buffered solution (PBS). An inverted microscope was utilized to visualize wound healing at 0, 24, 48, and 72 h. Wound-healing assays were conducted in triplicate.

### Cell migration and invasion assays

For migration assays, transfected cells were resuspended in 200 μL of serum-free RPMI 1640 medium and seeded onto transwell chambers (Corning, NY, USA) with 8-μm pore membranes (5 × 10^3^ cells/well). The chambers were then incubated in RPMI 1640 with 30% FBS at 37 °C in 5% CO_2_. After 48 h, the cells adhering to the chamber’s lower surface were fixed, whereas cells remaining on the upper surface were removed. After staining in a dye solution containing 0.5% crystal violet, the cells from three randomly selected high-power fields were counted under a microscope (Olympus, Tokyo, Japan). Migration assays were conducted in triplicate. For the invasion assay, the upper surface of the insert membrane was first coated with matrigel (BD Biosciences, San Jose, CA, USA). Transfected cells were cultured into the upper chamber with serum-free medium, and the lower chamber was filled with medium containing 30% FBS. After 48 h incubation, the cells adhering to the lower surface were fixed with methanol, stained with crystal violet and counted under a microscope. The cells were counted under a microscope in three randomly selected fields. Each sample was performed three times.

### miR-1236-3p target gene prediction

We predicted potential direct common target genes of miR-1236-3p using miRNA databases including TargetScan [22], miRanda [23], and miRDB [24]. At the intersection of three databases, the MTA2 gene had a lower mirSVR score and was selected as a prediction target gene.

### Protein extraction and western blotting

Cells were washed with PBS and radio-immunoprecipitation assay (RIPA) buffer (Beyotime, Shanghai, China) was utilized to extract total protein in accordance with the manufacturer’s instruction. After detecting the protein concentration, 40 μg of protein was loaded onto the sodium dodecyl sulfate polyacrylamide gel electrophoresis (SDS-PAGE) and then transferred to a polyvinyl fluoride membrane. The membranes were blocked with 5% non-fat milk in TBST for 1.5 h at room temperature and incubated overnight at 4 °C with the respective primary antibodies. The primary antibodies used were as follows: β-actin (1:1000, Absin Bioscience Inc, USA), MTA2 (1:500, Novus, USA), anti-E-cadherin (1:1000, CST, USA), anti-vimentin (1:1000, CST, USA), anti-N-cadherin (1:1000, CST, USA), anti-p-AKT (Ser 473) (1:1000, CST, USA), and anti-AKT (1:1000, CST, USA). After washing, membranes were incubated with the goat anti-rabbit secondary antibody (Beyotime, Shanghai, China) at room temperature for 2 h. Finally, blots were detected using an enhanced chemiluminescence system (Millipore Corporation, Temecula, CA, USA).

### Luciferase reporter assays

For the luciferase reporter assays, miR-1236-3p mimics, scramble, and the wild-type or mutant 3′-UTR of MTA2 were co-transfected into H-293T cells for 48 h. Luciferase activity was measured using the Dual-Luciferase Reporter Assay System (Promega, Madison, WI, USA). Renilla luciferase was used for normalization. Each sample was performed three times.

### Statistical analyses

All experiments were performed at least three times. Prism 6.07 software (GraphPad, Inc., La Jolla, CA, USA) and SPSS 19.0 (SPSS Inc., Chicago, IL, USA) was used to conduct the statistical analysis. The data are presented as the mean ± SD, and Student’s t test was performed for comparisons between two groups. *P *<  0.05 was considered statistically significant.

## Results

### miR-1236-3p is downregulated in GC cell lines and specimens

**Fig. 1:**
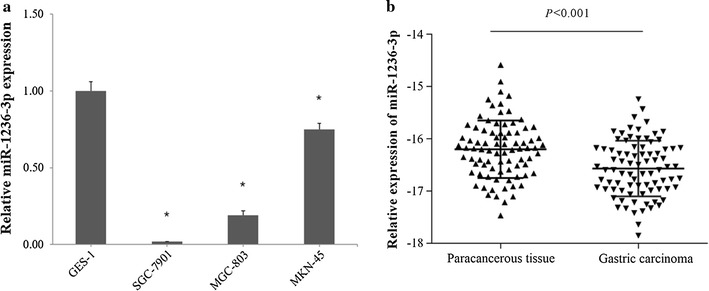
The expression level of miR-1236-3p in gastric cancer tissues and cells lines. **a** qRT-PCR analysis of miR-1236-3p expression in three human GC cell lines and one normal cell line. **b** qRT-PCR analysis of miR-1236-3p expression in human GC tissue samples and their matched normal adjacent tissues from 83 GC patients. *P < 0.05

To examine miR-1236-3p expression in GC cells, we measured the levels of miR-1236-3p in three GC cell lines, MKN-45, SGC-7901, and MGC-803, as well as a normal human gastric cell line, GES-1. The results indicated that the expression of miR-1236-3p was downregulated in MKN-45, SGC-7901, and MGC-803 cells compared with expression in GES-1 cells (Fig. 1a). Moreover, we measured the miR-1236-3p expression levels in 83 pairs of GC tissues and their adjacent normal tissues, and found that their levels were decreased in GC tissues compared with non-cancerous tissues (Fig. 1b). We also analyzed the associations between the miR-1236-3p expression level and the clinicopathological parameters of GC patients. The patients were divided into two groups. The cancer tissues with higher expression of miR-1236-3p than their adjacent normal tissues were selected as the high group, while those with less expression of miR-1236-3p than their adjacent normal tissues were selected as the low group. The results indicated that a low miR-1236-3p expression level was correlated with high TNM stage (*P* = 0.003), lymph node metastasis (*P* = 0.018), and differentiation (*P* = 0.015). These data suggest that miR-1236-3p might act as a tumor suppressor in GC (Table 1).

### miR-1236-3p suppresses GC cell proliferation

**Fig. 2:**
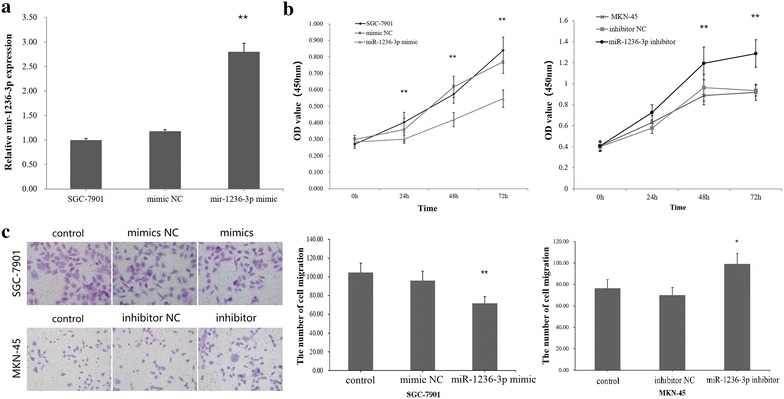
miR-1236-3p inhibited GC cells proliferation and migration. **a** The expression of miR-1236-3p in SGC-7901 cells transfected with miR-1236-3p mimic or negative control (NC). **b** The CCK-8 cell proliferation assays were detected at 0, 24, 48, 72 h after transfection. **c** Transwell migration assays of SGC-7901 cells transfected with miR-1236-3p mimic or NC, and MKN-45 cells transfected with miR-1236-3p inhibitor or NC. *P < 0.05, **P < 0.01

To investigate the function of miR-1236-3p in GC cells, miR-1236-3p mimics and inhibitor were transfected into SGC-7901 or MKN-45 cells respectively. miR-1236-3p expression was confirmed with qRT-PCR after transfection (Fig. 2a). CCK-8 cell proliferation assay displayed that cell proliferation was inhibited in SGC-7901 cells transfected with miR-1236-3p mimics, but enhanced in MKN-45 cells transfected with miR-1236-3p inhibitor (Fig. 2b). These results suggested that miR-1236-3p could efficiently inhibit GC cell proliferation.

### miR-1236-3p inhibits GC cell migration and metastasis

**Fig. 3:**
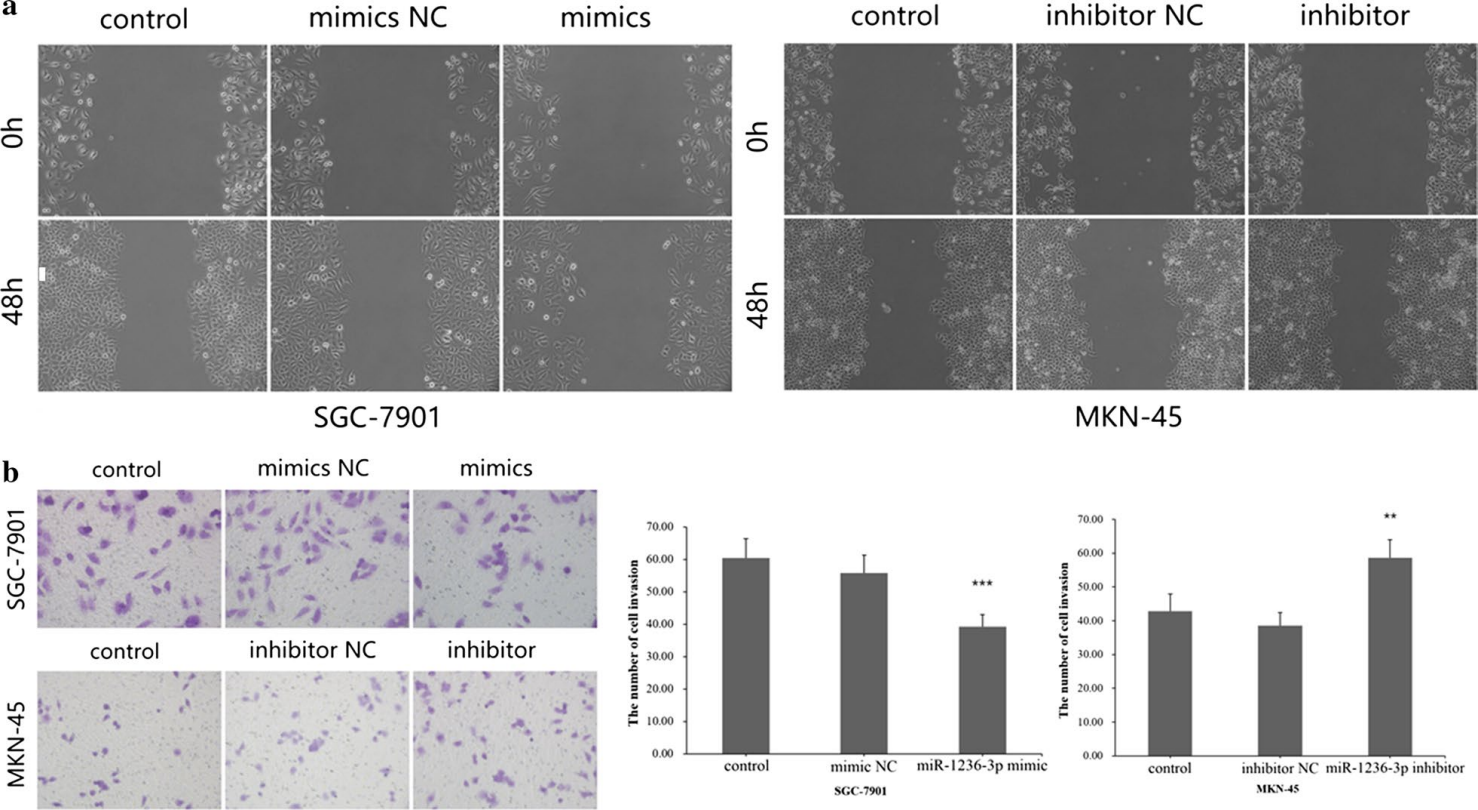
miR-1236-3p suppressed wound healing and cell invasion in gastric cancer. **a** The cells were treated as indicated above, wound-healing assays were used to detect the cell migration. **b** Cell invasion was detected using Transwell assays. **P < 0.01, ***P < 0.001

To analyze the migration and invasion functions of miR-1236-3p in GC cell, we performed trans-well migration assays using SGC-7901 and MKN-45 cells transfected with either miR-1236-3p mimics or inhibitor. The results showed that miR-1236-3p overexpression inhibited GC cell migration, and downregulation of miR-1236-3p expression had the opposite effect (Fig. 2c). Consistently, decreased cell migration was also observed when miR-1236-3p was upregulated in wound healing assays (Fig. 3a), suggesting that miR-1236-3p can suppress migration. Furthermore, trans-well invasion assays suggested that miR-1236-3p overexpression significantly suppressed GC cell invasive capacity, as showed in Fig. 3b. Taken together, these findings indicate that miR-1236-3p can inhibit GC cell migration and metastasis.

### MTA2 is a direct common target of miR-1236-3p

**Fig. 4:**
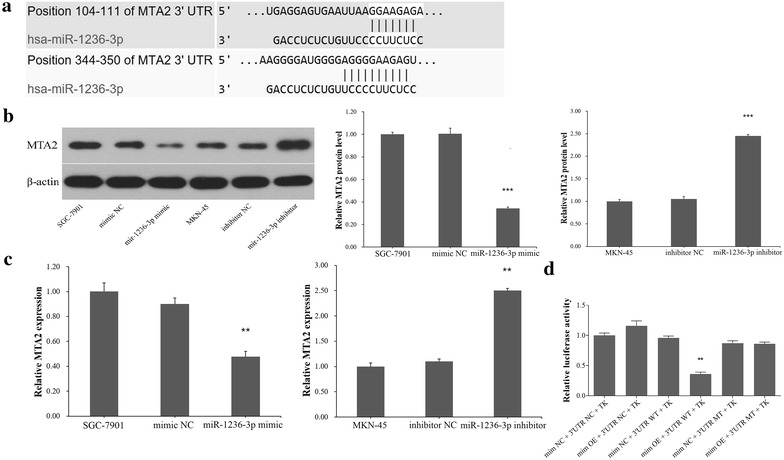
miR-1236-3p inhibited MTA2 expression by targeting its 3′-UTR. **a** Two predicted miR-1236-3p binding sites located in the MTA2 3′-UTR. **b** The protein expression level of MTA2 was measured by Western blotting assay. **c** The mRNA expression level of MTA2 was determined by real-time PCR. **d** Relative luciferase activity of MTA2 in wild-type or mutant. **P < 0.01, ***P < 0.001

**Fig. 5:**
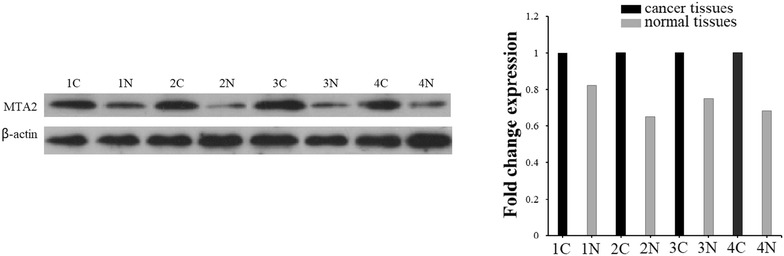
miR-1236-3p inhibited MTA2 expression in gastric cancer tissues. The protein level of MTA2 in four gastric cancer patients was measured

To explore the molecular mechanism by which miR-1236-3p contributes to GC progression, three computational algorithms, TargetScan, miRDB, and miRanda, were used in combination to search for potential miR-1236-3p targets. Among the candidates, MTA2, a potent oncogene that is frequently upregulated in human cancers [25, 26], was predicted to be a miR-1236-3p target by all three of the algorithms and was selected for further experimental verification. The predicted interaction between miR-1236-3p and the target site in the MTA2 3′-UTR is illustrated in Fig. 4a. The 3′-UTR of MTA2 contains two conserved binding sites for miR-1236-3p. Therefore, we selected MTA2 for further investigation, and measured its expression in GC cell lines at both the mRNA and protein levels. To investigate whether miR-1236-3p impacted MTA2 expression, we examined MTA2 protein levels in SGC-7901 cells transfected with miR-1236-3p mimics and MKN-45 cells transfected with miR-1236-3p inhibitor, the results showed that MTA2 was downregulated when miRNA was upregulated, and upregulated when miR-1236-3p was downregulated (Fig. 4b). To determine the level at which miR-1236-3p regulate MTA2 expression, we examined the expression of MTA2 mRNA after transfection. Upregulation or downregulation of miR-1236-3p had the same effects on MTA2 mRNA levels as proteins levels (Fig. 4c). These results demonstrated that miR-1236-3p regulates MTA2 protein expression and mRNA decay at the post-transcriptional level. Next, we employed a luciferase reporter system to confirm whether MTA2 was directly regulated by miR-1236-3p. The wild-type or mutant 3′-UTR of the MTA2 mRNA was inserted into a luciferase reporter vector, and then each construct was co-transfected with miR-1236-3p mimics. We observed that miR-1236-3p inhibited the luciferase reporter activity of the wild-type MTA2 3′-UTR, but did not significantly change the luciferase reporter activity of the 3′-UTR with mutated binding sites (Fig. 4d). Moreover, we detected the expression level of MTA2 protein in four miR-1236-3p downregulated GC tissues. The result showed that MTA2 level in gastric cancer tissues were higher than that in adjacent tissues (Fig. 5). These results indicate that MTA2 is a direct common target of miR-1236-3p.

### miR-1236-3p suppressed GC cell EMT

**Fig. 6:**
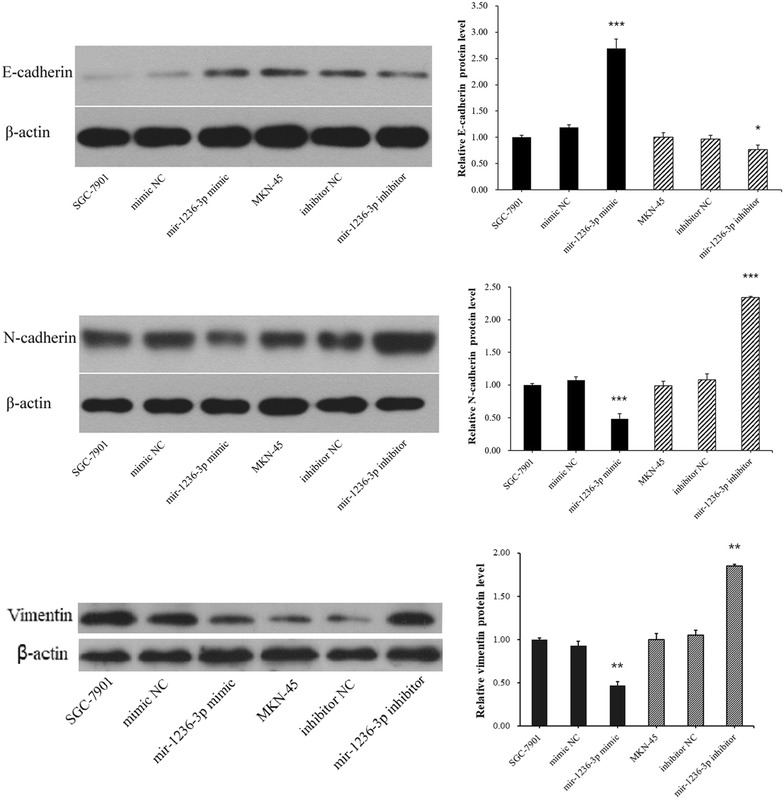
The protein expression levels of E-cadherin, vimentin and N-cadherin were measured by Western blotting assay. *P < 0.05, **P < 0.01, ***P < 0.001

**Fig. 7:**
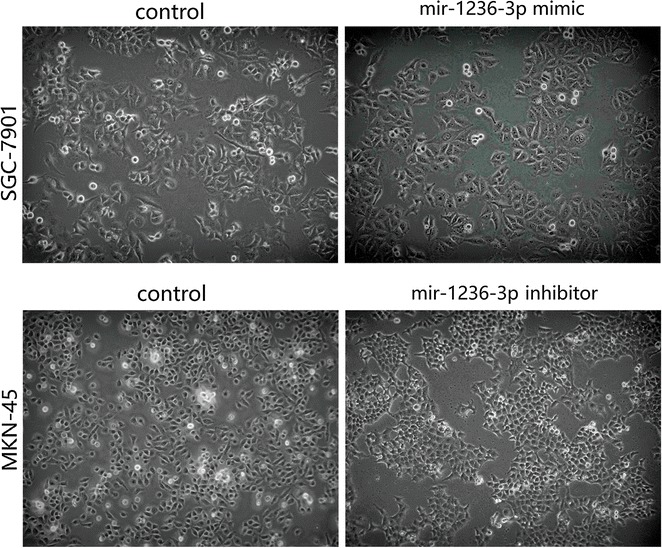
The morphological changes in SGC-7901 and MKN-45 cells after transfection

The foregoing findings suggested miR-1236-3p may participate in GC invasion progression. Hence, we speculated that miR-1236-3p might be associated with the EMT of gastric cells to GC. To evaluate the possibility that miR-1236-3p might regulate the EMT, we transfected SGC-7901 and MKN-45 cells with miR-1236-3p mimics or the inhibitor respectively, and then examined the expression of E-cadherin, vimentin and N-cadherin using western blotting. We found that E-cadherin level was increased, but vimentin and N-cadherin levels was reduced when miR-1236-3p were over-expressed. Furthermore, the E-cadherin level was reduced but vimentin and N-cadherin levels was increased when miR-1236-3p was downregulated (Fig. 6). We also observed that miR-1236-3p upregulation resulted in morphological changes from an extended morphology to more organized cell–cell contacts in SGC-7901 cells, and miR-1236-3p downregulation had the opposite result in MKN-45 cells (Fig. 7). Therefore, these results showed that miR-1236-3p suppressed GC cell EMT.

### miR-1236-3p inhibits the Akt signaling pathway in GC cells

**Fig. 8:**
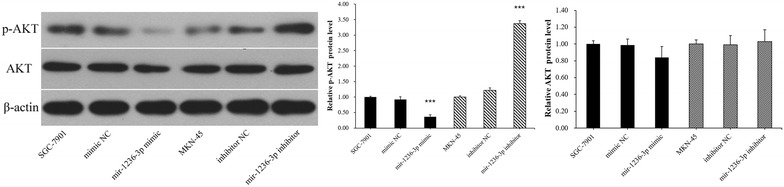
The protein expression levels of AKT and p-AKT were determined by Western blotting assay. ***P < 0.001

Previous studies have suggested that the miR-1236-3p target gene MTA2 is involved in Akt signaling pathway regulation [27]. In the present study, we verified the influence of miR-1236-3p on the protein levels of AKT and p-AKT. Our results showed that high levels of miR-1236-3p decreases the phosphorylation of AKT and low levels of miR-1236-3p increases the phosphorylation of AKT (Fig. 8). Collectively, our results revealed that miR-1236-3p suppressed the Akt signaling pathway partly through its target MTA2.

## Discussion

Previous studies have indicated that miRNAs can function as tumor suppressors or promoters in human cancers. For example, miR-203 could suppress the proliferation and metastasis of hepatocellular carcinoma by targeting ADAM9 [28]. miR-592 functioned as a tumor suppressor in lung cancer by targeting SOX9 [29]. miR-92a promoted cell proliferation and invasion by targeting FBXW7 [30]. miR-519d could promote melanoma progression by downregulating EphA4 [31], among other mechanisms. Recent studies revealed that miR-1236-3p played a critical role in human cancers. It functioned as a tumor suppressor in hepatocellular carcinoma, ovarian carcinoma, renal cell carcinoma, bladder cancer, lung cancer, and breast cancer [17–21]. In this study, we examined the roles of miR-1236-3p in GC. miR-1236-3p down-regulation was observed in GC tissues specimens and cell lines. Moreover, miR-1236-3p down-regulation was clearly relative to advanced clinical stage. To further explore the function of miR-1236-3p in GC, we performed a series of functional analyses. These experiments demonstrated that up-regulation of miR-1236-3p significantly inhibited the cell proliferation and reduced the number of migrated and invaded cells, and down-regulation of miR-1236-3p promoted cell proliferation and increased the number of migrated and invaded cells. These results suggested a tumor suppressor role for miR-1236-3p in GC progression. To explore the potential mechanism of miR-1236-3p in GC, the miR-1236-3p candidate target genes were predicted with TargetScan, miRanda, and miRDB, and MTA2 was selected as a potential target gene for miR-1236-3p. MTA2 is a member of the metastasis-associated gene family that has been reported to be closely associated with tumor progression [25]. MTA2 participated in the initiation and progression of a variety of tumors [32–34], and MTA2 over-expression promoted the proliferation and invasion of GC cells [26, 35]. MTA2 over-expression in ERa-positive breast cancer cells resulted in an enhanced anchorage-independent growth [36]. This evidence provided support to the hypothesis that MTA2 could act as an oncogene in many cancers. In our study, luciferase assays were performed and MTA2 was identified as a novel target of miR-1236-3p. In addition, we found that MTA2 mRNA and protein levels were negatively related to miR-1236-3p in GC cells, indicating that miR-1236-3p can negatively regulate MTA2 expression in GC. EMT is an early stage of tumor invasion and metastasis. In this process, cell morphology changes from cobblestone to spindle, cell surface adhesion molecules decrease, cell adhesion ability decreases, invasion and migration capacity increases, and metastasis of cancer cells is promoted. In this study, we found that after overexpression of miR-1236-3p, the cell morphology showed an epithelialization trend. After inhibition of miR-1236-3p, the cell morphology showed a tendency of mesenchymal cells. And we demonstrated that miR-1236-3p inhibited EMT process in GC cells by up-regulating E-cadherin expression, and inhibited the expression levels of vimentin and N-cadherin, but down-regulated miR-1236-3p produced the opposite effects. We then investigated whether mir-1236-3p affected signaling pathways in GC cells. Several signaling pathways including the Wnt/β-catenin and PI3K/Akt pathways have been reported to be aberrantly activated in the progression of GC [37, 38]. Our study revealed that miR-1236-3p over-expression decreases the phosphorylation of AKT and inhibition of miR-1236-3p increases the phosphorylation of AKT. Thus, these results suggested that miR-1236-3p inhibited the cell invasion by partly suppressing the PI3K/Akt signaling pathway. In summary, our results showed that miR-1236-3p functioned as a tumor suppressor to inhibit cell invasion in GC by targeting MTA2 and inhibiting the EMT process. The miR-1236-3p-MTA2 axis provides insight into the mechanisms underlying tumor metastasis, and may serve as a promising therapeutic target for GC treatment.

## Conclusions

We demonstrated that miR-1236-3p and MTA2 showed an inverse expression pattern in GC and that their functional roles in the development of GC were exerted by regulating EMT and PI3K/Akt signaling. Hence, these results suggested that miR-1236-3p and MTA2 were considered as new molecular biomarkers in predicting the aggressive biology of GC and novel therapeutic targets for GC.

## Abbreviations

GC: gastric cancer
real-time PCR: real-time polymerase chain reaction
UTR: untranslated region
EMT: epithelial–mesenchymal transition
ORF: open reading frame
AFP: alpha-fetoprotein
NC: negative control
CCK-8: Cell Counting Kit-8
PBS: phosphate buffered solution
RIPA: radio-immunoprecipitation assay
SDS-PAGE: sodium dodecyl sulfate polyacrylamide gel electrophoresis
